# Careful medical interview and ultrasonography enabled detection of acute kidney injury and hematoma after lumbar trigger point injection—a case report

**DOI:** 10.1186/s40981-021-00416-0

**Published:** 2021-01-26

**Authors:** Satoshi Sato, Shunsuke Tachibana, Kayoko Okazaki, Hitoshi Namba, Takahiro Ichimiya, Michiaki Yamakage

**Affiliations:** 1 Department of Anesthesiology, Asahikawa City Hospital, 1-1-65 Kinseicho, Asahikawa, Hokkaido 070-8610 Japan; 2 Department of Anesthesiology, Sapporo Medical University School of Medicine, South 1, West 16, Chuo-ku, Sapporo, Hokkaido 060-8543 Japan

**Keywords:** Complication of lumbar trigger point injection, Kidney injury and hematoma, Ultrasonography, Aortic dissection

## Abstract

**Background:**

Trigger point blocks are now widely practiced, especially in pain treatment. Among the complications of lumbar trigger point injection, reports of medically induced kidney injury are very rare, and diagnosis during emergency treatment is rare.

**Case presentation:**

A 78-year-old woman on antiplatelet medication following a stroke was diagnosed with treatable type A aortic dissection at another hospital after undergoing lumbar trigger point injection. On arrival at our hospital, there were no signs of hemodynamic deterioration. Additional careful medical re-interview and ultrasonography by anesthesiologists enabled a definitive diagnosis of acute kidney damage and hematoma caused by lumbar trigger point injection, and aortic dissection surgery was abandoned.

**Conclusion:**

This clinical case demonstrates the importance of awareness of potential kidney injury and hematoma during lumbar trigger point injection.

## Background

Trigger point injection therapy is a common treatment for chronic pain in primary medicine and is in general use by family doctors and orthopedists for chronic low back pain. Although several complications have been reported for this technique [1], acute kidney damage and/or hematoma around the kidney have been previously described as complications of lumbar trigger point injection [2].

In this report, we present a case in which additional careful medical re-interview and ultrasonography enabled a definitive diagnosis of acute renal failure and hematoma caused by lumbar trigger point injection, which was subsequently treated conservatively.

## Case report

We obtained approval from the institutional ethics committee of Asahikawa City Hospital and written informed consent from the patient for publication of this case report. The study followed the CARE guidelines and the appropriate EQUATOR guidelines.

The patient was a 78-year-old-woman with a previous history of cerebral infarction and hyperlipidemia who was taking therapeutic aspirin (Bayaspirin, 100 mg) without other anticoagulants. She complained of uncomfortableness and a sudden, new onset of low back but no chest pain 30 minutes after receiving lumbar trigger point injection of lidocaine by her family doctor. She was referred to a primary emergency hospital with a suspicious diagnosis of aortic dissection. She was conscious with systolic blood pressure around 170 mmHg with a left-right difference of 30 mmHg on arrival at the hospital. Contrast-enhanced computed tomography (CT) revealed Stanford type A aortic dissection and she was transferred to our hospital. She remained conscious with stable vital signs throughout this time.

**Fig. 1:**
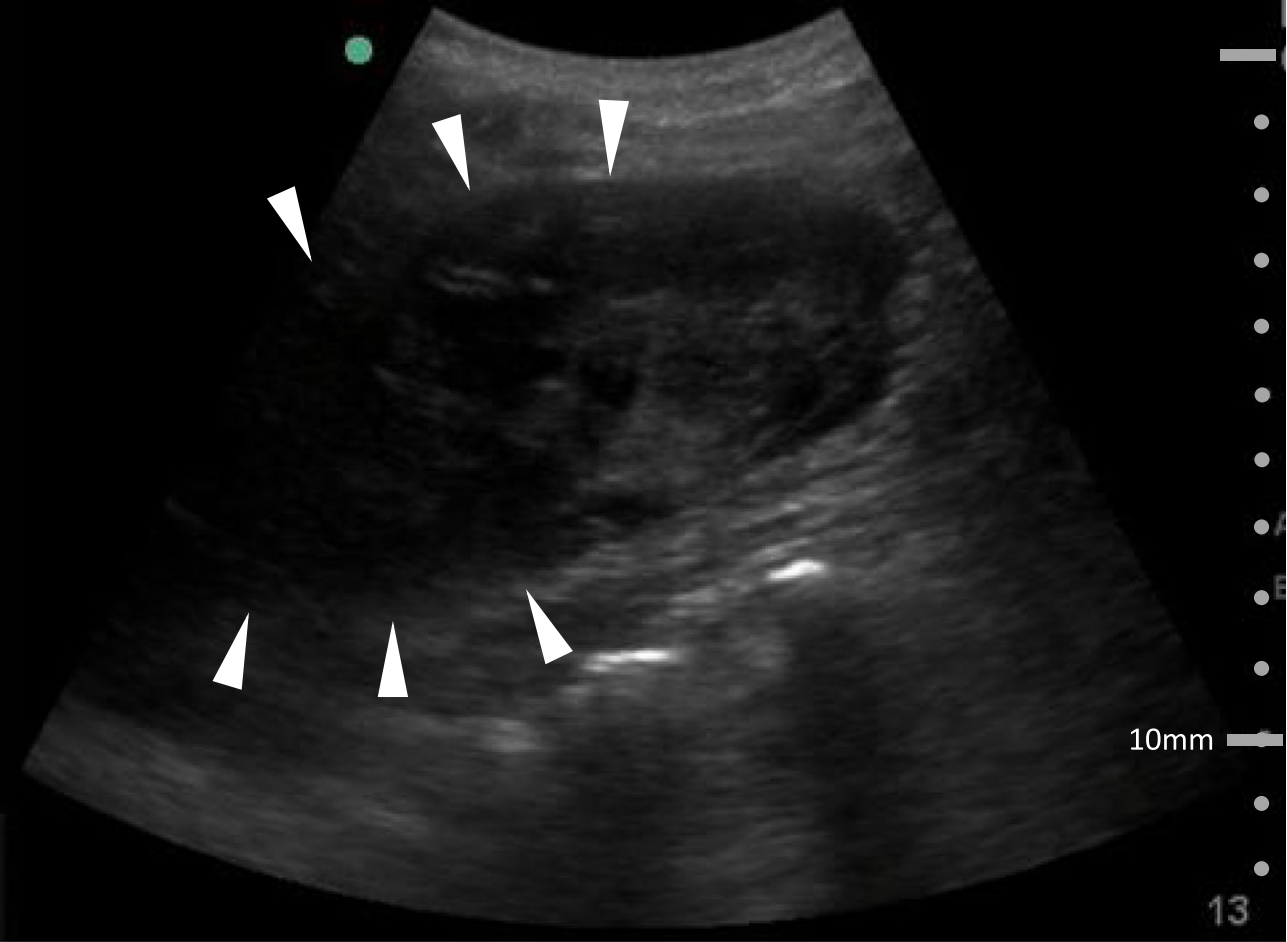
Ultrasound image of the left first lumbar level. Hypoechoic area (arrow head) suggestive of hematoma was detected below the trigger point injection site observed in the second emergency room. The renal parenchyma is a slightly hyperechoic area to the right of the renal hematoma

**Fig. 2:**
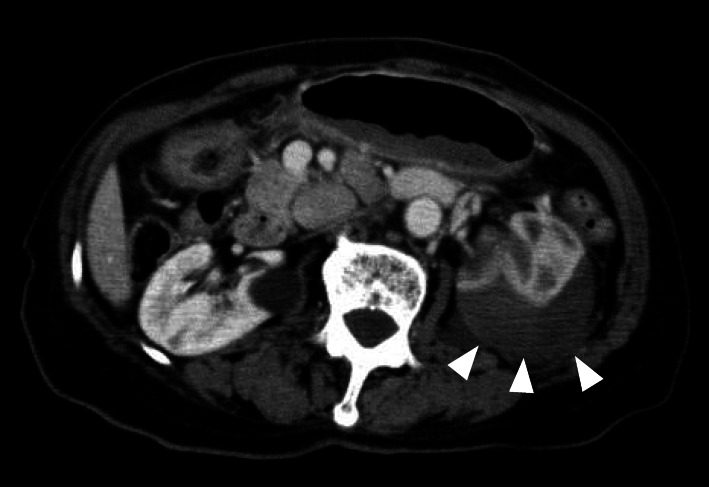
Plain CT image of the abdomen. Note the hematoma excluding the left kidney (arrow head)

Since the patient was scheduled for emergency surgery for type A aortic dissection, we noticed something unusual while conducting our additional detailed medical interview and physical examination in preparation for the surgery. Based on the interview, renal hematoma was suspected because of localized lumbar pain and a trigger block puncture mark in the same area. We suspected that her symptoms were related to the lumbar trigger point injection. Ultrasonography revealed a hypoechoic lesion displacing the left kidney, approximately 3 cm beneath the injection site, suggesting left renal hematoma caused by trigger point injection, as indicated by the white arrows in Fig. 1. CT examination confirmed these findings (Fig. 2), which further revealed a dissection lumen from the ascending aorta to the aortic arch occluded by a thrombus with a maximum short diameter of 55 mm. No obvious false lumen was seen distal to the aortic arch or stenosis in the ventral partial branches. After making the diagnosis of renal hematoma, drainage of the left kidney and surrounding hematoma was also noted. Because the dissection lumen of the aortic dissection was completely occluded by the thrombus, emergency surgery for type A aortic dissection was not performed.

Further investigations revealed no progression of anemia, no increase in blood staining on CT, and no active bleeding, and conservative therapy was initiated with antihypertensive treatment for the thrombotic obstructive type A aortic dissection. After diagnosis of the renal hematoma, aspirin administration was discontinued. Only non-steroidal anti-inflammatory analgesics and antibiotics were prescribed for the renal hematoma. She was discharged from the hospital without sequelae 2 weeks after admission.

## Discussion

Renal hematoma is a rare complication of lumbar trigger point block, although it is an important treatment in the pain clinic. We experienced a case in which careful medical re-interview and ultrasonography enabled a definitive diagnosis of acute kidney injury and hematoma caused by lumbar trigger point injection. Among the 276 claims associated with invasive procedures for chronic pain management in the American Society of Anesthesiologists Closed Claims Project [1], 17 involved trigger point injections; however, there are few reports of acute kidney injury or hematoma resulting from lumbar trigger point injections [2]. Serious complications reported after trigger point injection therapy include abscess, necrotizing fasciitis, osteomyelitis, and gas gangrene [3]. Non-infectious complications include pneumothorax [4], which is the most common non-infectious claim according to the American Society of Anesthesiologists Closed Claims Project [1], air embolism, and intrathecal injection presenting as hemiplegia [5].

Regarding the trigger point injection performed by the previous doctor, whether it was done blindly or with ultrasonography-guided technique, how long the needle was, and other detailed information was unknown because it was not reported to us. Therefore, we could not discuss the technique in this case. In the present case, the renal hematoma was a complication of trigger block injection and was not associated with aortic dissection; moreover, the hematoma appeared to be iatrogenic, caused by prolonged bleeding due to antiplatelet therapy. Non-traumatic renal hematoma can be classified into three types: internal, subcapsular, and perirenal [6]. Non-traumatic subcapsular hematoma can also be associated with renal tumors [7]. Hence, it is important to determine the history of a bruise on the abdomen or back and to rule out the presence of a tumor. Most iatrogenic cases occur secondary to extracorporeal shock wave lithotripsy for renal biopsy and renal stones. Secondary infection and hypertension are complications of renal hematoma. In the case of hypertension secondary to kidney injury, termed Page kidney injury, renin hypersecretion occurs in response to the renal hematoma.

Ultrasonography is useful for visualizing free fluid in the trauma setting but is inferior to CT in terms of resolution and ability to accurately characterize renal injury [8, 9]. It is unable to distinguish fresh blood from the extravasated urine and cannot identify vascular pedicle injuries or segmental infarct [7]. However, it can be used for follow-up on hydronephrosis, renal laceration managed non-operatively, and postoperative fluid collection [10]. The lack of ionizing radiation, which is one of the main advantages of ultrasonography, is very relevant for pediatric patients.

In the dorsal subcutaneous to renal anatomy, structures located at a thickness of approximately 3 cm beneath the subcutaneous tissue and fat include the latissimus dorsi, quadratus lumborum, and transverse abdominal muscles; the inferior surface of the diaphragm above the kidney; and Gerota’s fascia [11]. Between the transverse abdominal muscles and Gerota’s fascia are perirenal fatty tissues located inferiorly and laterally to the kidney. The kidneys are located between the 11th thoracic and 3rd lumbar vertebrae, with the left kidney higher than the right kidney. In addition, the lower pole of the kidney is located close to the skin, and for this reason, it is chosen as the site of needle puncture for the collection of renal biopsy tissue. In the present case, trigger block puncture was performed at the level of the left first lumbar vertebra. Because of its vulnerability to injury of the lower renal pole, great care must be taken when performing skin puncture in this area. Although the dorsal kidney does not contain large blood vessels, the kidneys have rich blood flow. To minimize the risk of kidney injury, and on the basis of the present case, it is considered that the only recommendation is to carefully perform trigger point block to prevent renal injury in patients receiving aspirin.

The present treatment strategy was conservative and included follow-up observation because renal capsular hematoma is sometimes accompanied by bleeding. Our careful re-interview and primary ultrasonography enabled accurate identification of the cause of bleeding in this patient and guided the course of appropriate treatment.

In conclusion, hematoma caused by lumbar trigger point injection is a relatively rare condition. Our case report highlights the importance of careful medical re-interview and ultrasonography at the primary medical examination.

## Data Availability

The data that support the findings of this report are the property of Asahikawa City Hospital and were provided to the authors under restricted conditions. Although not publicly available, the data can be obtained from the authors upon reasonable request, and with the prior permission of Asahikawa City Hospital.
